# Clinical impact of follow‐up endomyocardial biopsy in myocarditis during or after immune‐suppressive therapy

**DOI:** 10.1002/ehf2.15349

**Published:** 2025-06-30

**Authors:** Anna Baritussio, Andrea Silvio Giordani, Stefania Rizzo, Cristina Vicenzetto, Monica De Gaspari, Elisa Carturan, Giuseppe Toscano, Federico Scognamiglio, Giuseppe Tarantini, Sabino Iliceto, Cristina Basso, Renzo Marcolongo, Alida Linda Patrizia Caforio

**Affiliations:** ^1^ Cardiology, Department of Cardiac, Thoracic, Vascular Sciences and Public Health Padua University Hospital and University of Padua Padua Italy; ^2^ Cardiovascular Pathology, Department of Cardiac, Thoracic, Vascular Sciences and Public Health Padua University Hospital and University of Padua Padua Italy; ^3^ Cardiac Surgery, Department of Cardiac, Thoracic, Vascular Sciences and Public Health Padua University Hospital and University of Padua Padua Italy

**Keywords:** biopsy‐proven myocarditis, endomyocardial biopsy, myocarditis, outcome, patients' management

## Abstract

**Aims:**

While the diagnostic role of endomyocardial biopsy (EMB) in myocarditis is unquestioned, little is known about its indications and clinical value during long‐term follow‐up. We aim to report our experience on the clinical relevance of repeating EMB in a cohort of biopsy‐proven myocarditis patients treated with immune‐suppressive therapy (IMT).

**Methods and results:**

We retrospectively included 92 patients with virus‐negative EMB‐proven myocarditis treated with IMT, of whom 22 [73% male, 37 years, interquartile range (IQR) 33–48] received at least one, uneventful, follow‐up EMB, 3.5 years (IQR 2.5–5.3) after the first one. Follow‐up EMB was performed because of clinical worsening (*n* = 12) or suspected myocarditis relapse (*n* = 7) and to assess IMT response (*n* = 3). Patients receiving follow‐up EMB more likely had abnormal troponin levels (*P* = 0.048) and a trend towards lower prevalence of anti‐heart auto‐antibodies positivity at diagnosis (*P* = 0.05) and showed worse imaging findings at follow‐up. Active or borderline myocarditis on follow‐up EMB was found in 12 patients, leading to a change in IMT regimen in 10 (83%); among patients with evidence of healed myocarditis, 2 had a change in IMT regimen, 2 underwent heart transplant evaluation check list, 2 had mitral valve treatment, 1 received ventricular tachycardia ablation and 1 primary prevention implantable cardioverter‐defibrillator implantation.

**Conclusions:**

One‐fourth of EMB‐proven myocarditis patients had a clinical indication to repeat EMB at least once during long‐term follow‐up. Follow‐up EMB was safe in all cases; it showed active or borderline myocarditis in 55% of patients and lead to a change in management in the majority of patients.

## Graphical abstract

Results and clinical implication of repeat endomyocardial biopsy (EMB).

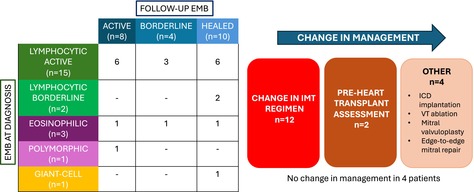

## Introduction

Endomyocardial biopsy (EMB) is the gold standard for diagnosis of certainty and etiological identification of suspected myocarditis, which is a heterogeneous disease in terms of clinical presentation, disease course and late outcome.^1^, ^2^, ^3^ The indications to EMB, recently updated by the American, European and Japanese heart failure societies,^4^ now include hemodynamically stable patients with clinically suspected myocarditis, along with the long‐standing indication in patients with fulminant presentation, suspected myocarditis presenting with cardiogenic shock or acute heart failure (HF) and left ventricular dysfunction, with or without malignant ventricular arrhythmias and/or conduction abnormalities. According to the most recent European guidelines on HF,^5^ the identification of virus‐negative biopsy‐proven myocarditis should prompt patients' assessment to identify those eligible to immune‐suppressive therapy (IMT) that has shown to be prognostically efficacious, both in terms of clinical and functional features, in different recent studies.^6^, ^7^, ^8^

The relevance of EMB is unequivocal for myocarditis diagnosis^9^, ^10^, ^11^ and for rejection surveillance in heart transplant recipients.^12^, ^13^, ^14^ Less is known about its role at follow‐up in patients with biopsy‐proven myocarditis on chronic IMT; only few, relatively dated studies and case reports have explored the contribution of follow‐up EMB in myocarditis, early in the disease course and soon after initiation of IMT.^15^, ^16^, ^17^, ^18^ To the best of our knowledge, there is no published detailed analysis on long‐term follow‐up EMB as a guide to IMT in biopsy‐proven myocarditis. We therefore report our experience on the clinical relevance of repeating EMB in a cohort of patients with biopsy‐proven myocarditis receiving IMT.

## Materials and methods

We retrospectively included in the analysis biopsy‐proven myocarditis patients receiving IMT, derived from a single‐centre registry of almost 1000 myocarditis patients (of whom 358 are biopsy‐proven) on active follow‐up at Padua University Hospital (Padua, Italy).

Biopsy‐proven myocarditis was defined based on the 2013 European Society of Cardiology (ESC)^1^ and World Health Organization's^1^, ^18^, ^19^ criteria, by established histological (Dallas criteria, histological evidence of inflammatory infiltrates within the myocardium associated with myocyte degeneration and necrosis of non‐ischaemic origin), immunological and immunohistochemical (≥14 leucocytes/mm^2^ including up to 4 monocytes/mm^2^ with the presence of CD 3 positive T‐lymphocytes ≥7 cells/mm^2^) criteria.

Immune‐suppressive treatment was initiated, under the supervision of a clinical immunologist, according to the 2013 ESC consensus^1^ on myocardial and pericardial diseases and the 2021 ESC guidelines on HF^5^ and was carefully personalized to patients' characteristics, as previously described.^8^

Among the enrolled patients, we focused our analysis on the characteristics of those receiving at least one follow‐up EMB. Follow‐up EMB was performed by obtaining 4–6 myocardial samples, 1–2 mm in size, only from the right ventricle, by trans‐femoral or trans‐jugular approach^1^, ^4^, ^9^; one or two frozen EMB specimens per patient were used for polymerase chain reaction (PCR) and reverse transcriptase PCR analysis for detection of cardiotropic viruses' genome simultaneously to histological analysis. The indications to follow‐up EMB, based on the clinician's discretion, followed international consensus^4^ and can be grouped into three main categories: clinical worsening, suspected myocarditis relapse and assessment of response to IMT (as clinically indicated). Clinical worsening was defined as an unexplained decline in echocardiographic left ventricular systolic function at follow‐up, with or without HF signs/symptoms and/or unexplained ventricular arrhythmias, in the absence of coronary artery disease (ruled out by coronary angiography or coronary computed tomography angiography) or any other cardiac condition that could have explained the clinical findings.^1^ Suspected myocarditis relapse was defined according to ESC/WHO criteria^1^, ^18^, ^19^ for suspected myocarditis. Findings on follow‐up EMB were classified as follows: (a) active and (b) borderline myocarditis (ESC/WHO criteria^1^, ^18^, ^19^), when showing inflammatory infiltrates with or without (in borderline cases) myocytes degeneration or necrosis, and (c) healed myocarditis, when showing resolution of both myocardial inflammation and necrosis, with or without non‐ischaemic myocardial fibrosis (this may include post‐myocarditis dilated cardiomyopathy, *restitutio ad integrum* of the myocardium, endomyocardial fibrosis, etc.).

A thorough discussion was performed with each patient, explaining the risks related to follow‐up EMB as well as its expected clinical benefits, as was done for the first, baseline, EMB.

Clinical, demographic, histologic, laboratory and imaging characteristics at diagnosis were collected, along with clinical and imaging features at follow‐up. The implications on patients' management following repeat EMB were assessed to determine the clinical impact of this procedure during follow‐up. The study was approved by the local Ethical Committee (protocol number 0062881).

Categorical data were expressed as *n* (%), continuous data as median [interquartile range (IQR)]. Continuous variables were compared by two‐tailed unpaired *t*‐test (for normally distributed data sets) or by Mann–Whitney *U* test, as appropriate; categorical variables were compared by chi‐square test or by Fisher's exact test, as appropriate. A *P* value <0.05 was considered statistically significant. Data sets were analysed using Jamovi (Sydney, Australia, version 2.5).^20^

## Results

### Characteristics of biopsy‐proven patients treated with IMT

Out of 358 biopsy‐proven myocarditis cases, we included 92 patients (26%, 46 years, IQR 32–56, 59% male) who were treated with IMT. Patients' characteristics are reported in *Table* 1. Two thirds of patients (70%) had active lymphocytic myocarditis on first EMB, 8% had borderline lymphocytic, 12% eosinophilic, 7% giant‐cell, 1 patient polymorphic myocarditis and 1 had cardiac sarcoidosis.

**Table 1:** Patients' characteristics.

	All (*n* = 92)	Follow‐up MB (*n* = 22)	No follow‐up EMB (*n* = 70)	*P* value
Gender, male	54 (59)	16 (73)	38 (54)	0.14
Age at diagnosis, years	46 (32–56)	37 (33–48)	46 (30–58)	0.35
FHx of ID	13 (14)	2 (10)	11 (16)	0.50
FHx of heart disease	33 (37)	11 (52)	22 (31)	0.15
FHx of CAD	14 (18)	5 (24)	9 (13)	0.31
FHx of DCM	6 (8)	2 (10)	4 (6)	0.64
FHx of myocarditis	1 (1)	0	1 (1)	1.0
Acute viral infection before myocarditis diagnosis	22 (24)	5 (24)	17 (24)	1.0
ID	33 (36)	6 (27)	27 (39)	0.34
History of myocarditis	18 (20)	2 (10)	16 (23)	0.23
Arterial hypertension	17 (19)	2 (10)	15 (21)	0.34
Diabetes	6 (7)	1 (5)	5 (7)	1.0
Clinical presentation				
Infarct‐like	25 (27)	7 (32)	18 (26)	0.58
Heart failure	52 (57)	10 (45)	42 (60)	0.23
Arrhythmias	9 (10)	3 (14)	6 (9)	0.44
Asymptomatic	6 (7)	2 (10)	4 (6)	0.63
Fulminant presentation	8 (9)	2 (10)	6 (9)	1.0
NYHA class at diagnosis				0.47
I	36 (39)	11 (50)	25 (36)	
II–IV	56 (61)	11 (50)	45 (64)	
Sinus rhythm at presentation	82/89 (92)	22 (100)	60 (89)	0.49
Bundle branch block at presentation	17/81 (21)	2 (10)	15 (25)	0.42
AHA positivity	52/80 (65)	10/21 (47)	42/59 (71)	0.05
LVEDV at diagnosis, mL/m^2^	91 (72–116)	93 (72–114)	89 (72–116)	0.86
LVEF at diagnosis, %	35 (26–50)	36 (28–54)	35 (36–48)	0.73
FAC at diagnosis, %	34 (25–40)	38 (34–48)	29 (24–39)	0.07
Abnormal TnI at diagnosis	48/69 (70)	12 (92)	36 (64)	0.048
LVEF on CMR at diagnosis	26 (21–46)	44 (21–53)	26 (22–44)	0.29
RVEF on CMR at diagnosis	46 (35–56)	46 (34–55)	53 (41–57)	0.21
Oedema on CMR at diagnosis	30/69 (44)	9 (53)	21 (40)	0.68
LGE on CMR at diagnosis	51/68 (75)	13/18 (72)	39/50 (76)	0.64
Treatment				
Beta‐blockers	69/79 (87)	17/19 (89)	52/60 (87)	1.0
Amiodarone	9/79 (11)	5/19 (26)	4/60 (7)	0.03
ACE inhibitors	36/79 (46)	8/19 (42)	28/60 (47)	0.79
Sartans	21/72 (29)	6/19 (32)	15/53 (28)	0.59
IMT				0.82
PDN + AZA	55 (60)	15 (68)	40 (57)	
PDN + MMF	5 (5)	1 (5)	4 (6)	
PDN + MTX	6 (7)	2 (9)	4 (6)	
PDN + Cya	2 (2)	1 (5)	1 (1)	
PDN	12 (13)	2 (9)	10 (14)	
MMF	1 (1)	0	1 (1)	
MTX	1 (1)	0	1 (1)	
Other combinations	10 (11)	1 (5)	9 (13)	
IMT duration, days	610 (332–799)	638	610	0.66
Second line IMT, %	36 (40)	13 (59)	23 (33)	0.03
Second line IMT				0.64
PDN + AZA	2 (6)	1 (8)	1 (4)	
PDN + MMF	20 (56)	9 (69)	11 (48)	
PDN + MTX	2 (6)	0	2 (9)	
PDN + Cya	2 (6)	0	2 (9)	
PDN	3 (8)	1 (8)	2 (9)	
MMF	2 (6)	0	2 (9)	
Other combinations	5 (14)	2 (15)	3 (13)	
Positive genetic testing	14/47 (30)	7/18 (39)	7/29 (24)	0.28
NYHA class at follow up				0.06
I	67/82 (82)	20/20 (100)	47/62 (76)	
II, III	15/82 (18)	0	15/62 (24)	
Duration of follow up, months	60 (31–90)	73 (47–92)	55 (25–86)	0.045
LVEDV at follow‐up, mL/m^2^	69 (58–85)	81 (64–89)	66 (56–78)	0.02
LVEF at follow‐up, %	54 (46–60)	51 (44–56)	54 (50–62)	0.08
FAC at follow‐up, %	43 (39–50)	40 (36–45)	44 (40–50)	0.05
Abnormal TnI at follow‐up	11/51 (22)	6 (40)	5 (14)	0.06
Death/heart transplant	11 (12)	2 (9)	9 (13)	1.0

Abbreviations: ACE, angiotensin converting enzyme; AHA, anti‐heart auto‐antibodies; AZA, azathioprine; CAD, coronary artery disease; CyA, cyclosporine A; DCM, dilated cardiomyopathy; FAC, fractional area change; FHx, family history; ID, immune disease; IMT, immune‐suppressive treatment; LGE, late gadolinium enhancement; LVEDV, left ventricular end‐diastolic volume; LVEF, left ventricular ejection fraction; MMF, mycofenolate; MTX, methotrexate; NYHA, New York Heart Association; PDN, Prednisone; RVEF, right ventricular ejection fraction; TnI, Troponin I.

More than half of patients presented with HF; eight patients had a fulminant presentation. Echocardiographic left ventricular systolic function was overall moderately impaired on echocardiography at diagnosis while right ventricular function was overall only mildly impaired. Almost two thirds of patients undergoing CMR at diagnosis had findings consistent with acute/subacute (52%) or healed myocarditis (5%); findings consistent with DCM were reported in 28% of cases, an ischaemic necrosis was reported in 2% of patients and Takotsubo cardiomyopathy in 5%. Patients received IMT mainly for worsening/unremitting HF, with a tailored approach as previously described,^8^ for median 1.7 years; one third of patients received a second line IMT. One fourth of patients (36%) were implanted with an implantable cardioverter‐defibrillator (ICD). At follow‐up (median duration 60 months, IQR 31–90 months), four patients underwent heart transplantation, and seven patients died. Most of the patients were in NYHA class I at the last available follow‐up and overall showed preserved biventricular function on echocardiogram.

### Characteristics of patients receiving follow‐up EMB

Twenty‐two patients (24%) underwent a follow‐up EMB without procedural complications (7, 32%, through a jugular approach), 3.5 years (IQR 2.5–5.3) following the first EMB (*Table* 2). The reasons to repeat EMB were clinical worsening (*n* = 12, unexplained left ventricular dysfunction with/without signs of HF in 10 cases, ventricular arrhythmias in 2 cases), clinically suspected myocarditis relapse (*n* = 7) and assessment of response to IMT (*n* = 3); five patients received follow‐up EMB while still on IMT, and three patients received more than one EMB during follow‐up. Findings on follow‐up EMB were consistent with active myocarditis in eight cases (36%), borderline myocarditis in four cases and healed myocarditis in the remaining cases (45%) (*Figure*s 1 and 2). Active myocarditis was more commonly found among patients with active lymphocytic myocarditis at diagnosis (6/8). There was no difference in the frequency of active myocarditis at follow‐up EMB with regards to the time course between first and follow‐up EMB (*P* = 0.74).

**Table 2:** Characteristics of patients undergoing follow‐up endomyocardial biopsy.

	Gender	Age	Clinical presentation	Baseline LVEF echo	EMB results	PCR on EMB	CMR results	AHA	Genetic test	Arrhythmias	ICD	Treatment	IMT (duration)	Reason to repeat EMB (time to EMB)	FU EMB results	Results of repeat CMR	Change in management	LVEF last FU
PT1	Male	47	HF	20%	Lymphocytic borderline myocarditis	Neg	DCM without LGE	Pos OS	TNNT2	NSVT	No	BB, ARNi,furosemide, MRA, amiodarone	AZA + PDN (24 months)	HF (5 years)	Healed myocarditis	DCM without LGE	CPT, HTx check list	39%
PT2	Male	33	ACS‐like	20%	Lymphocytic active myocarditis	Neg	DCM with LGE	Neg	TTN	NSVT	No	BB, ARNi, MRA	AZA (3 months) + PDN (13 months)	Relapse (3.1 years)	Lymphocytic active myocarditis	DCM	Re‐start of IMT (CyA + PDN)	38%
PT3	Male	48	HF, SVT	28%	Lymphomocytic active myocarditis	Neg	DCM without LGE	Pos OS	Not tested	NSVT	No	BB, Amiodarone, ACEi	AZA + PDN (26 months)	HF (6.9 years)	Healed myocarditis	Hypokinetic cardiomyopathy without LGE	HF OMT	46%
PT4	Male	18	ACS‐like	55%	Lymphocytic active myocarditis+	Neg	Acute myocarditis	Pos OS	DSP	NSVT	No (ILR)	Sotalol	AZA + PDN (9 months) then MMF + PDN (10 months)	Relapse (3.9 years)	Lymphocytic borderline myocarditis	‐	Re‐start of IMT (MMF + PDN)	53%
PT5	Female	35	HF	31%	Lymphocytic active myocarditis	Neg	Acute myocarditis	Neg	Neg	No	No	BB, sartans	PDN + AZA (24 months)	Active inflammation on CMR (2.4 years)	Healed myocarditis	Acute myocarditis	—	54%
PT6	Female	37	ACS‐like	68%	Lymphocytic active myocarditis	Neg	Eosinophilic myoc	Neg	Neg	No	No	BB, MRA, furosemide	AZA + PDN (24 months)	HF (2.4 years)	Healed myocarditis (endomyocardial fibrosis)	Endomyocardial fibrosis	Mitral valvuloplasty	56%
PT7	Male	47	SVT	38%	Giant‐cell myocarditis	Neg	Acute myocarditis	Pos OS	Neg	SVT	Yes	BB, ACEi, Amiodarone Mexitletine	PDN + Cyclosporine+MMF (5 months) then AZA	SVT (1.5 years)	Healed myocarditis	—	VT ablation	42%
PT8	Male	33	HF	18%	Lyphocytic active myocarditis	Neg	‐	Pos OS, AIDA	Not tested	No	Yes	BB, sartans	PDN + CyA (40 months)	HF (0.9 years)	Borderline myocarditis	DCM	IMT manteinance	51%
PT9	Male	37	ACS‐like	67%	Lymphocytic active myocarditis	Neg	—	AIDA	Neg	ICD shock on VT	Yes	BB, ACEi	PDN + AZA (40 months)	NSVT (9.1 years)	Lymphocytic active myocarditis (PVB19)	—	—	56%
PT10	Male	67	HF	28%	Eosinophilic myocarditis	Neg	Acute myocarditis	Neg	Not tested	NSVT	No	BB, ACEi, MRA, amiodarone, furosemide	PDN + MTX (8 months), then MMF (24 months)	Right HF (4.7 years)	Healed myocarditis	—	Mepolizumab (asthma and eosinophilia)	25%
PT11	Male	14	ACS‐like	50%	Eosinophilic myocarditis	Neg	—	—	Neg	No	No	None	PDN + MTX (27 months)	Assessment of IMT response (0.8 years)	Borderline myocarditis	—	IMT maintenance	57%
PT12	Male	36	Arrhythmia	31%	Lymphocytic active myocarditis	Neg	Cardiac amyloidosis	Pos AIDA	Mybpc3	SVT	Yes	BB, ACEi	PDN + AZA(1mo), then MMF (22 mo) and CyA (5mo)	HF (3 years)	Lymphocytic active myocarditis (replicating PVB19)	—	IMT withdrawal	33%
PT13	Female	51	HF	52%	Lymphocytic active myocarditis	Neg	Chronic myocarditis	AIDA	Not tested	NSVT	No	BB, sartans	PDN + AZA (29 months), then MMF	HF (5.2 years)	Healed myocarditis	—	ICD, HF OMT	36%
PT14	Male	46	Asymptomatic	38%	Lymphocytic active myocarditis	Neg	Acute myocarditis	Pos OS	Neg	No	No	BB, ACEi	PDN + AZA (17 months) then MMF	Arrhythmias, LV dysfunction (4.8 years)	Active myocarditis	—	Restart PDN + MMF	54%
PT15	Male	32	HF	25%	Lymphocytic active myocarditis	Neg	Acute myocarditis	Neg	TTN	No	Yes	BB, ACEi	PDN + AZA (2 years), then MMF (17 months)	LV dysfunction (3.5 years)	Healed myocarditis	DCM with non‐ischaemic fibrosis	CPT, HTx check list	41%
PT16	Female	55	Asymptomatic	37%	Lymphocytic active myocarditis	Neg	—	Pos OS, AIDA	Neg	No	No	BB, ARNi, MRA	PDN + MMF (29 months), then AZA	LV dysfunction (8.4 years)	Lymphocytic borderline myocarditis	—	Re‐start of IMT (PDN + MMF)	35%
PT17	Male	48	HF	20%	Lymphocytic active myocarditis	Neg	Subacute myocarditis	Neg	Neg	No	Yes	BB, sartans	PDN + AZA (29 months)	HF (2.8 years)	Healed myocarditis	—	—	45%
PT18	Male	26	Arrhythmias	54%	Lymphocytic borderline myocarditis	Neg	Acute myocarditis	Pos OS	Neg	No	Yes	BB, ACEi	PDN + AZA (30 months), then PDN + MMF (24 months)*	Persistent Troponin release and LV dysfunction (11.4 years)	Healed myocarditis	—	IMT withdrawal	48%
PT19	Female	40	HF	23%	Lymphocytic active myocarditis	Neg	—	Pos OS, AIDA	Neg	SVT	Yes	BB, amiodarone	PDN + AZA (7 months) then MMF (25 months)	Relapse (2.5 years)	Active Lymphocytic myocarditis**	—	MitraClip	46%
PT20	Female	32	HF	35%	Lymphocytic active myocarditis	Neg	Acute myocarditis	Neg	TTN	NSVT	Yes	BB, sartans	PDN + AZA (8 months)	Relapse (12.6 years)	Active myocarditis	—	Re‐start of IMT (PDN + AZA)	52%
PT21	Male	26	SCA‐like	66%	Polymorphic myocarditis	Neg	Acute myocarditis	Neg	DSP	No	No	BB, ACEi	PDN + AZA (18 months)	Relapse (1.4 years)	Lymphocytic active myocarditis	Chronic myocarditis	PDN + MMF	59%
PT22	Male	81	SCA‐like	60%	Eosinophilic myocarditis	Neg	—	Neg	Neg	No	No	Amiodarone, warfarin	PDN (18 months)	Relapse (2.9 years)	Active eosinophilic myocarditis	—	Mepolizumab	45%

Abbreviations: ACS, acute coronary syndrome; AIDA, anti‐intercalated disk auto‐antibodies; ARNi, Angiotensin Receptor‐Neprilysin Inhibitor; BB, beta‐blocker; CMR, cardiovascular magnetic resonance; CPT, cardio‐pulmonary test; DSP, desmoplakin; HF, heart failure; HTx, heart transplant; LV, left ventricle; MRA, mineralocorticoid receptor antagonist; Mybpc3, cardiac myosin‐binding protein C; NSVT, non‐sustained ventricular tachycardia; OMT, optimal medical therapy; OS, organ specific; PT, patient; TNT, titin; TNNT2, troponin T2; SVT, sustained ventricular tachycardia; VT, ventricular tachycardia. For the remaining abbreviations, see *Table* 1

* Two cycles, each of 2 years duration, due to LV dysfunction and persistent troponin release.

** Focal myocarditis, IMT contraindicated ad follow‐up due to recurrent infections.

**Figure 1:**
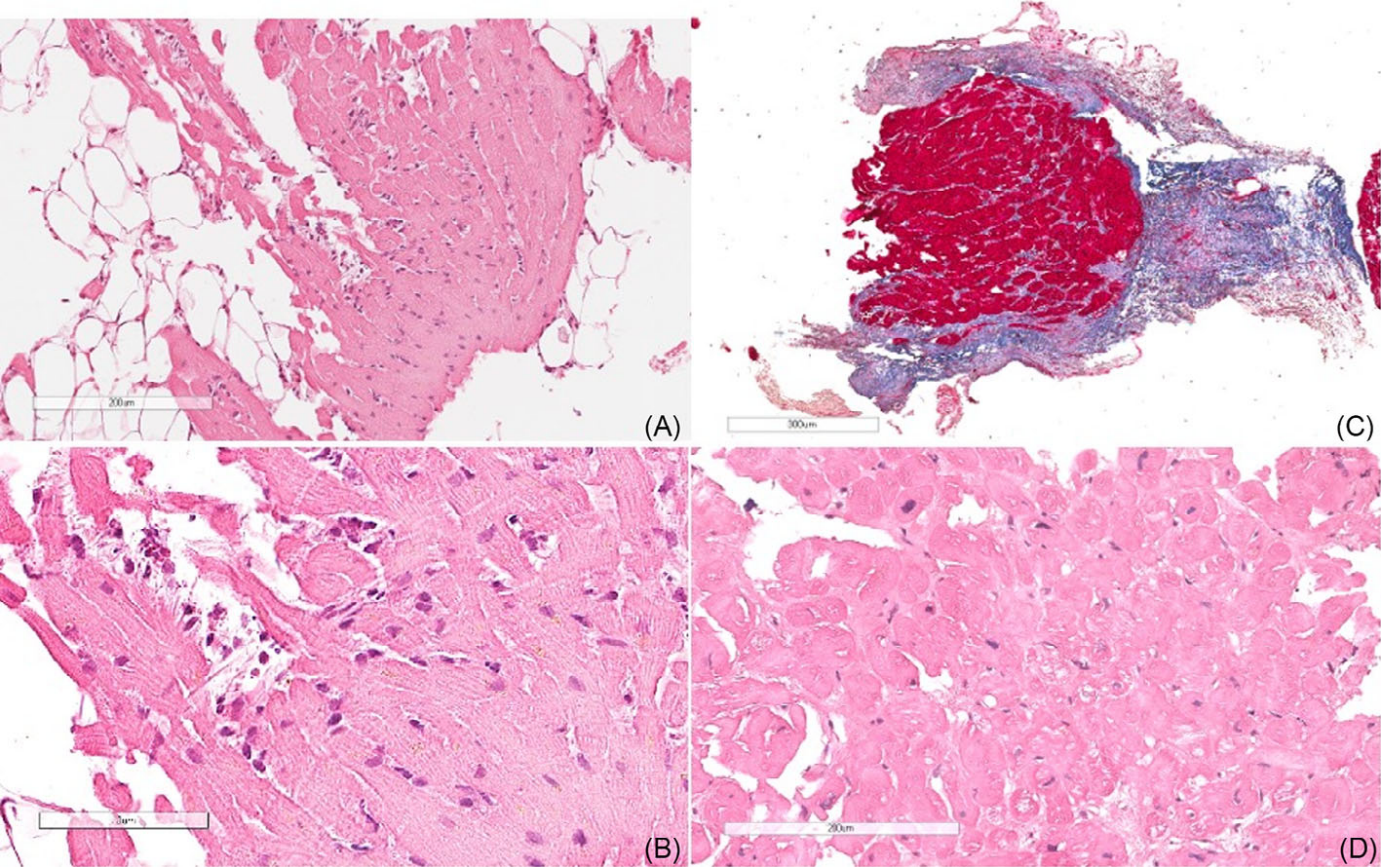
First endomyocardial biopsy (EMB) (A). At low magnification, inflammatory infiltrates mostly consist of eosinophils with myocyte necrosis, in keeping with eosinophilic myocarditis [haematoxylin–eosin, (A) scale bar 200 μm, (B) scale bar 70 μm; close‐up in B]. Repeat EMB (5 years later) (C). At low magnification, note the endocardial fibrous thickening, in keeping with endomyocardial fibrosis (Azan trichrome, scale bar 300 μm). At higher magnification, no evidence of inflammation and necrosis, but cardiomyopathic changes, with dysmetric nuclei and vacuolization (D) (haematoxylin–eosin, scale bar 200 μm).

**Figure 2:**
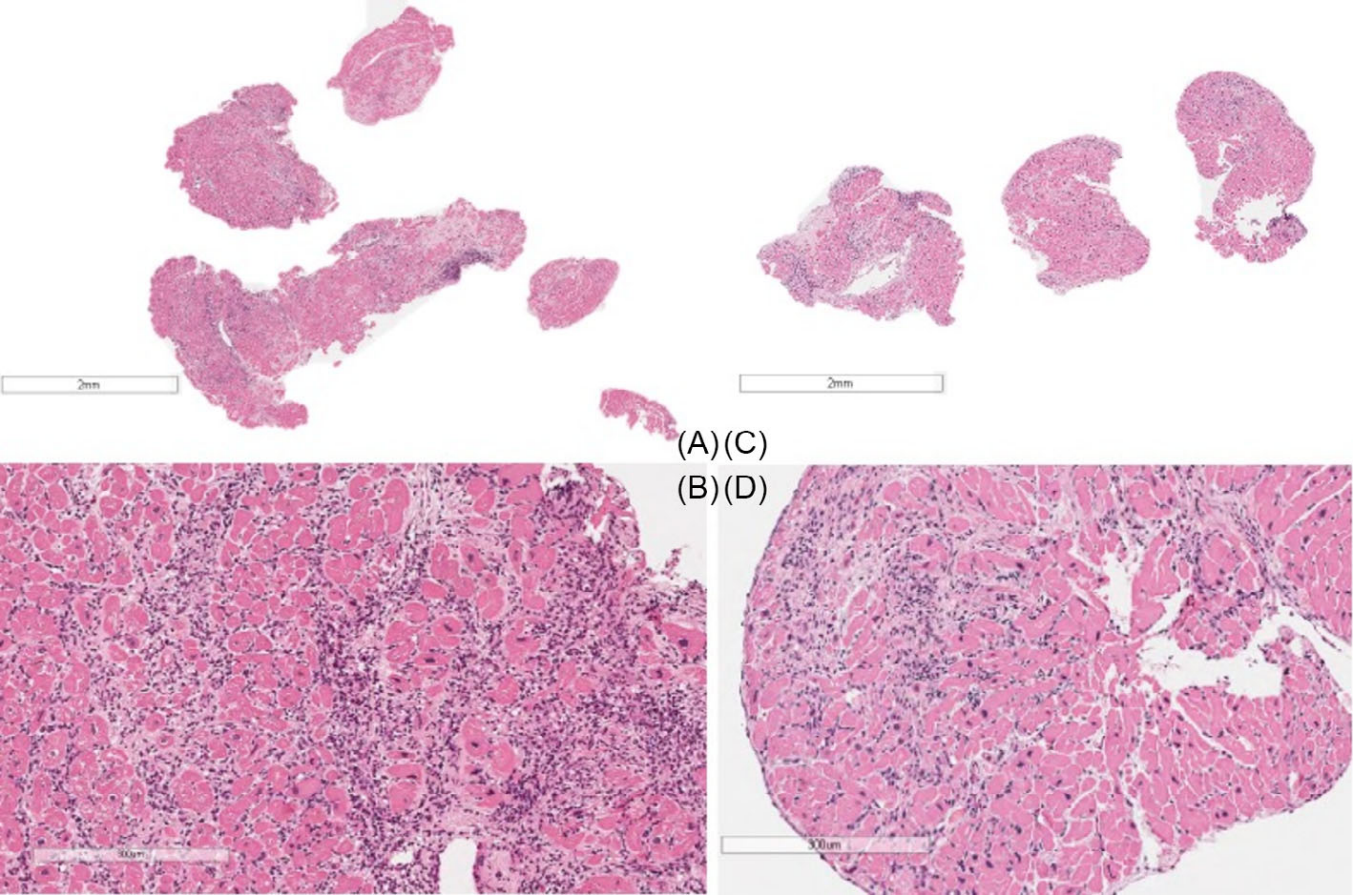
First EMB (A). Panoramic view showing five endomyocardial samples, all involved by dense and diffuse lymphocytic infiltrate also in aggregate, without giant cells (haematoxylin–eosin, scale bar 2 mm). (B) At higher magnification, the inflammatory infiltrates are associated with massive necrosis of cardiomyocytes with dysmetric nuclei and cardiomyopathic changes, in keeping with diffuse active lymphocytic myocarditis and cardiomyopathy (haematoxylin–eosin, scale bar 300 μm). Repeat EMB (3 years later) (C). Panoramic view of three endomyocardial samples, showing diffuse lymphomonocytic inflammatory infiltrates (haematoxylin–eosin, scale bar 2 mm), associated with myocyte necrosis, replacement type fibrosis and cardiomyopathic changes. The final diagnosis was relapsing lymphocytic myocarditis in cardiomyopathy (haematoxylin–eosin, scale bar 300 μm).

Patients receiving follow‐up EMB did not differ significantly from those not receiving a follow‐up EMB with regards to demographic characteristics and cardiovascular risk factors, clinical presentation and imaging findings at diagnosis. At the time the decision was made to repeat EMB, follow‐up CMR data were only available in eight patients (*Table* 2); discordant findings were found in three cases, showing active myocardial inflammation on follow‐up EMB and findings consistent with DCM (*n* = 2) and chronic myocarditis (*n* = 1) on follow‐up CMR. Patients receiving follow‐up EMB were however more likely to have abnormal troponin levels at diagnosis (92% vs. 64%, *P* = 0.048) and showed a trend towards lower prevalence of anti‐heart auto‐antibodies positivity at diagnosis as compared with patients not receiving follow‐up EMB (47% vs. 71%, *P* = 0.05). They were also more likely on anti‐arrhythmic treatment with amiodarone while there was no difference in the remaining standard medical treatment between the two groups; there was also no difference in the first line of IMT between the two groups, but a second line IMT was more frequent among patients receiving a follow‐up EMB. Duration of follow‐up was longer in patients undergoing follow‐up EMB, which showed significantly lower right ventricular function (40% vs. 44%, *P* = 0.05), larger left ventricular volumes (81 mL/m^2^ vs. 66 mL/m^2^, *P* = 0.02) and a trend towards lower left ventricular function at last available follow‐up.

### Clinical implications of repeating EMB at follow‐up

Follow‐up EMB led to a change in IMT in 55% of patients: ongoing IMT regimen was maintained in two patients with evidence of borderline myocarditis on follow‐up EMB; IMT was re‐started or IMT regimen was changed in eight patients (5 with active and 2 with borderline myocarditis; 1 patient was started on Mepolizumab due to asthma and persistent eosinophilia after excluding viral genome replication on follow‐up EMB, showing findings consistent with healed myocarditis) and IMT was withdrawn in two patients (because of evidence of active viral myocarditis with viral genome replication in 1 case) (Graphical abstract). Following the diagnosis of healed myocarditis on follow‐up EMB, a check list for heart transplant evaluation was started in two patients with advanced worsening HF. One patient with severe mitral regurgitation (MR) as a result of endomyocardial fibrosis (confirmed on follow‐up EMB) was treated with mitral annuloplasty while another one with severe functional MR due to post‐myocarditis DCM was treated with transcatheter edge‐to‐edge repair. One patient undergoing follow‐up EMB because of sustained ventricular tachycardia (VT) underwent VT ablation following histologic evidence of healed giant cell myocarditis; one patient received primary prevention ICD implantation following exclusion of potentially reversible processes on follow‐up EMB.

## Discussion

The main findings of our study were that (a) almost one fourth of biopsy‐proven myocarditis patients, with ongoing or previous IMT, had a clinical indication, during long‐term follow‐up, to repeat EMB; (b) follow‐up EMB was free from periprocedural complications in all cases and (c) showed active or borderline myocarditis in 55% of patients, leading to a change in IMT regimen in most of them; (d) patients receiving follow‐up EMB more likely had abnormal troponin levels at diagnosis and a trend towards lower frequency of anti‐heart auto‐antibodies positivity at diagnosis and showed worse imaging findings at last follow‐up.

### Myocarditis patients receiving follow‐up EMB

To the best of our knowledge, this is the first report on the long‐term role of integrating follow‐up EMB (median time from first EMB 3.5 years) in the diagnostic and therapeutic management of patients with biopsy‐proven myocarditis treated with IMT. In our cohort, 24% of biopsy‐proven patients treated with IMT received a follow‐up EMB. Although based on the clinician's discretion, indication to repeat EMB always followed international recommendations and was mainly due to clinical worsening during follow‐up, by means of decline in left ventricular function (12/22) and evidence of ventricular arrhythmias (2/22); this is in line with a previous study reporting that the main indications to repeat EMB in a large cohort of patients receiving the first EMB because of suspected myocarditis were indeed unexplained HF and VT.^17^

Our patients receiving repeat EMB showed worse clinical status during follow‐up, but we found no difference in clinical presentation and imaging findings at diagnosis as compared to patients not receiving follow‐up EMB. However, patients receiving follow‐up EMB more likely had abnormal troponin levels at diagnosis and were also more frequently on anti‐arrhythmic treatment; these features may reflect the fact that patients receiving repeat EMB during follow‐up were in fact more likely to have a worse clinical ‘phenotype’ at diagnosis. We also found a trend towards lower prevalence of anti‐heart auto‐antibodies positivity at diagnosis among patients receiving follow‐up EMB. We previously found that, before the introduction of IMT for the treatment of virus‐negative EMB‐proven myocarditis, anti‐heart auto‐antibodies positivity identified myocarditis patients with worse outcome^21^; as follow‐up EMB was not performed in the entire cohort, the association between anti‐heart auto‐antibodies positivity and outcome could not be explored in the present study.

Patients undergoing repeat EMB showed worse imaging findings at follow‐up, however reporting a better functional status, likely due to the intensified pharmacological treatment of their worsening clinical and functional status (i.e., treatment implemented with ARNI and SGLT2i).

In a previous study, patients with myocarditis on repeat EMB were more likely to have borderline myocarditis on first EMB (4/6 vs. 0/22, *P* = 0.0007)^17^; conversely, in our study, none of the two patients with borderline myocarditis on first EMB showed persistent myocardial inflammation on follow‐up EMB while active myocarditis on repeat EMB was more commonly found among patients with active lymphocytic myocarditis at diagnosis (6/8). This difference may be explained by the different time range between first and repeat biopsy, which was significantly shorter in the study by Dec et al. (on average 31 ± 6 days vs. median 3.5 years in our study).

### Clinical implications of follow‐up EMB

Histological diagnosis of myocarditis is key to guide treatment: according to the most recent European guidelines on the management of HF, patients with virus‐negative biopsy‐proven myocarditis should undergo at least 6–12 months of immunosuppressive treatment.^5^ This follows evidence from two randomized clinical trials and a recent propensity‐weighted study,^6^, ^7^, ^8^ along with single‐centre observational experiences, all showing the beneficial effect of IMT in virus‐negative biopsy‐proven myocarditis in terms of improvement in left ventricular function and reduction of adverse events at follow‐up. The TIMIC follow‐up study has also reported relapsing biopsy‐proven virus‐negative myocarditis in 6% of patients at long term follow‐up, clinically manifesting as decline in left ventricular function and showing cardiac function recovery following another 6‐month trial of IMT.^7^ One of the major clinical motivations to repeat EMB in our cohort was indeed to identify patients potentially benefitting from another course of IMT, and actually eight patients receiving repeat EMB from our cohort were prescribed another course of IMT, with improved functional and clinical status at follow‐up in five of them.

Searching for viral genomes on EMB, through PCR, is as well pivotal for patients' treatment,^1^, ^8^ as IMT in patients with viral myocarditis may not be beneficial, as shown in previous studies,^22^, ^23^ while also being of potential harm in cases of active viral replication, especially if cardiotropic viruses with direct pathogenic effect are identified (i.e., enterovirus or high load PVB19). Indeed, based on the most recent long‐term European registry of paediatric and adult myocarditis, although the direct pathogenic role of PVB19 and HHV6 is still debated, some investigators have suggested that high viral load and active viral replication differentiate a pathogenic infection from an innocent bystander viral genome presence.^24^ In one case from our cohort, the evidence of active myocardial viral replication on follow‐up EMB indeed led to IMT withdrawal to prevent potential direct cardiac damage and further worsening of clinical and functional status.

Aside from determining a change in IMT in all patients with active/borderline myocarditis, identifying healed myocarditis (therefore ruling out potentially reversible causes) on repeat EMB also had an implication on patients' management, for example by initiating a long‐term programme for advanced HF treatment (i.e., ICD implantation, check list initiation for heart transplant, etc.).

In our cohort, only a minority of patients had a follow‐up CMR prior to or at the time of follow‐up EMB, with discordant findings found in 3/8 patients; in two patients, evidence of active myocarditis on EMB, not confirmed on CMR, led to a change in IMT. Cardiovascular magnetic resonance is considered the non‐invasive gold standard for diagnosing myocarditis,^1^, ^25^ but its agreement with EMB varies widely according to the type of myocarditis (greatest discrepancy for borderline myocarditis^26^) and its clinical presentation (lower CMR's diagnostic accuracy for HF and arrhythmic presentation^27^). While Lake Louise criteria have shown high diagnostic accuracy in pseudo‐infarct presentation, tissue changes occurring during a longer disease course (i.e., oedema reabsorption, prevalence of myocytes apoptosis, rather than necrosis) make standard CMR sequences less likely to be diagnostic in HF and arrhythmic presentation.^26^, ^27^ Increasing evidence confirms that EMB and CMR are not interchangeable and that their agreement is less likely in chronic presentations. Therefore, CMR should be used as a complimentary test to EMB (potentially also serving as a guide to EMB), but it should neither delay EMB^1^ nor discourage its performance, if clinically indicated, when providing negative results.

## Past evidence and future perspectives

Only few studies assessed the role of repeat EMB in myocarditis patients, mainly addressing histological and functional changes in response to IMT, early after its initiation.^15^, ^16^, ^17^ Despite some variability in the duration of persistent myocardial inflammation (ongoing myocarditis was found in 40% of cases after 11 weeks of IMT in one study,^16^ complete resolution of histological features of myocarditis occurred 14 months after the first EMB in another study^15^), there was not a significant association with systolic function: Dec et al.^16^ reported improved systolic function in similar proportions of patients with ongoing versus resolved myocarditis on repeat EMB; similarly, Keogh et al.^15^ found normalized systolic function 48 h after IMT initiation in a patient with lymphocytic myocarditis presenting with cardiogenic shock, in spite of persistent histological features of myocarditis for up to 14 months after the first EMB.

The largest experience on follow‐up EMB derives from heart transplant recipients, where it is performed to rule out clinically suspected or silent acute rejection and to assess response to changes in IMT regimen. Despite wide differences in surveillance protocols, both in terms of number of repeat EMB and duration of histological surveillance following transplantation, studies have shown the greatest benefit of repeating EMB in transplanted patients with a clinical indication (i.e. clinical suspicion of acute rejection).^12^, ^13^, ^14^, ^28^, ^29^, ^30^

In keeping with this, our findings show that, when performed based on clinical indication, repeating EMB during long‐term follow‐up carries direct and favourable implications on patients' management. There is currently no evidence‐based consensus on the optimal long‐term follow‐up of myocarditis patients, although the American College of Cardiology has recently released some guidance on the clinical and imaging surveillance of symptomatic (stage C) and advanced (stage D) myocarditis patients.^31^ As CMR shows variable agreement with EMB, especially in chronic presentations, repeating EMB during long‐term follow‐up should follow the same indications as baseline EMB. Our study was not designed to identify features predicting which patients will need and benefit most from follow‐up EMB; therefore, further studies are warranted. The identification of these high‐risk patients will be pivotal to define future diagnostic algorithms for follow‐up EMB prior to IMT termination; moreover, it might allow the definition of dedicated follow‐up programmes integrating clinical, imaging and histological data.

## Limitations

Follow‐up EMB was not performed systematically in the entire cohort, but it was offered to selected patients based on the clinicians' discretion. This may represent a selection bias, as it is likely that patients with more severe disease course were offered a follow‐up EMB and we cannot exclude that the clinical impact of repeating EMB may therefore have been overestimated in our cohort. However, our series represents real‐life data from a large, single‐centre registry of myocarditis patients, and it may serve as a basis for multicentre studies to further assess the clinical implications of surveillance EMB in myocarditis patients and to address the need and applicability of a structured programme for follow‐up EMB in dedicated patients.

There is ongoing discussion on the diagnostic accuracy and clinical and prognostic correlates of borderline myocarditis, specifically when small foci of inflammatory cells are found in the absence of myocyte necrosis.^32^, ^33^ However, this definition was used in our study as it is yet part of the current diagnostic criteria,^1^, ^31^, ^34^ and it also reflects the wide time span covering baseline and follow‐up EMB.

Assessing the long‐term impact of changing patients' management following repeat EMB was beyond the scope of the manuscript, and the sample size and variable follow‐up duration after repeat EMB do not allow drawing definite conclusions; however, we reported a higher incidence of arrhythmias at follow‐up (although not reaching statistical significance) among patients not eligible to change in IMT based on repeat EMB results. Further studies are needed to clarify the best candidate to repeat EMB and the most appropriate timing for the greatest clinical benefit of follow‐up EMB.

## Conclusions

Almost one fourth of virus‐negative biopsy‐proven myocarditis patients receiving immune‐suppressive treatment had a clinical indication, during long‐term follow‐up, to repeat EMB, that was safe in all, showed active or borderline myocarditis in 55% and led to a change in management in the majority of cases.

## Funding

A.L.P.C. is funded by the European Union ‐ Next Generation EU ‐ NRRP M6C2 ‐ Investment 2.1 ‘Enhancement and strengthening of biomedical research in the NHS’ (project title: Biopsy‐proven paediatric and adult giant cell and other rare immune‐mediated forms of myocarditis: creation of a prospective multicentre Italian registry and a biobank network to identify clinical, immune and genetic predictors of dismal prognosis, relapse and response to immunosuppressive therapy; code PNRR‐MR1‐2022‐12375693, Cup: I93C22000560006). Views and opinions expressed are however those of the author(s) only and do not necessarily reflect those of the European Union. Neither the European Union nor the European Commission can be held responsible for them.

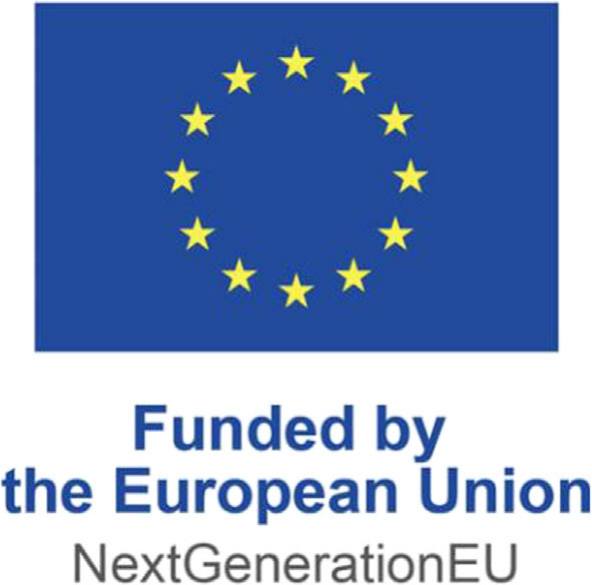

A.L.P.C. was also supported by the Italian Ministry of Health, Target Research, Rome, Italy, year 2019, RF‐2019‐12370183 (project title: *Biopsy‐Proven Myocarditis: Genetic Background, Predictors of Dismal Prognosis and of Response to Immunosuppressive Therapy and Preclinical Evaluation of Innovative Immunomodulatory Therapies*).

## Conflict of interest statement

None to declare.

## References

[1] Caforio AL , Pankuweit S , Arbustini E , Basso C , Gimeno‐Blanes J , Felix SB , *et al*. Current state of knowledge on aetiology, diagnosis, management, and therapy of myocarditis: a position statement of the European Society of Cardiology Working Group on Myocardial and Pericardial Diseases. Eur Heart J 2013;34:2636‐2648. doi:10.1093/eurheartj/eht210 23824828

[2] Tschöpe C , Ammirati E , Bozkurt B , Caforio ALP , Cooper LT , Felix SB , *et al*. Myocarditis and inflammatory cardiomyopathy: current evidence and future directions. Nat Rev Cardiol 2021;18:169‐193. doi:10.1038/s41569-020-00435-x 33046850 PMC7548534

[3] Basso C . Myocarditis. N Engl J Med 2022;387:1488‐1500. doi:10.1056/NEJMra2114478 36260793

[4] Seferović PM , Tsutsui H , Mcnamara DM , Ristić AD , Basso C , Bozkurt B , *et al*. Heart Failure Association, Heart Failure Society of America, and Japanese Heart Failure Society position statement on endomyocardial biopsy. J Card Fail 2021;27:727‐743. doi:10.1016/j.cardfail.2021.04.010 34022400

[5] McDonagh TA , Metra M , Adamo M , Gardner RS , Baumbach A , Böhm M , *et al*. 2021 ESC guidelines for the diagnosis and treatment of acute and chronic heart failure. Eur Heart J 2021;42:3599‐3726. doi:10.1093/eurheartj/ehab368 34447992

[6] Frustaci A , Russo MA , Chimenti C . Randomized study on the efficacy of immunosuppressive therapy in patients with virus‐negative inflammatory cardiomyopathy: the TIMIC study. Eur Heart J 2009;30:1995‐2002. doi:10.1093/eurheartj/ehp249 19556262

[7] Chimenti C , Russo MA , Frustaci A . Immunosuppressive therapy in virus‐negative inflammatory cardiomyopathy: 20‐year follow‐up of the TIMIC trial. Eur Heart J 2022;43:3463‐3473. doi:10.1093/eurheartj/ehac348 35831932 PMC9492235

[8] Caforio ALP , Giordani AS , Baritussio A , Marcolongo D , Vicenzetto C , Tarantini G , *et al*. Long‐term efficacy and safety of tailored immunosuppressive therapy in immune‐mediated biopsy‐proven myocarditis: a propensity‐weighted study. Eur J Heart Fail 2024;26:1175‐1185. doi:10.1002/ejhf.3220 38629741

[9] Cooper LT , Baughman K , Feldman AM , Frustaci A , Jessup M , Kuhl U , *et al*. The role of endomyocardial biopsy in the management of cardiovascular disease: a scientific statement from American Heart Association, the American College of Cardiology, and the European Society of Cardiology. J Am Coll Cardiol 2007;50:1914‐1931. doi:10.1016/j.jacc.2007.09.008 17980265

[10] Leone O , Veinot JP , Angelini A , Baandrup UT , Basso C , Berry G , *et al*. 2011 Consensus statement on endomyocardial biopsy from the Association for European Cardiovascular Pathology and the Society for Cardiovascular Pathology. Cardiovasc Pathol 2012;21:245‐274. doi:10.1016/j.carpath.2011.10.001 22137237

[11] Bozkurt B , Colvin M , Cook J , Cooper LT , Deswal A , Fonarow GC , *et al*. Current diagnostic and treatment strategies for specific dilated cardiomyopathies: a scientific statement from the American Heart Association. Circulation 2016;134:e579‐e646. doi:10.1161/CIR.0000000000000455 27832612

[12] Stehlik J , Starling RC , Movsesian MA , Fang JC , Brown RN , Hess ML , *et al*. Utility of long‐term surveillance endomyocardial biopsy: a multi‐institutional analysis. J Heart Lung Transplant 2006;25:1402‐1409. doi:10.1016/j.healun.2006.10.003 17178332

[13] Sethi GK , Copeland JC . Routine surveillance endomyocardial biopsy. Ann Thorac Surg 1997;64:1230. doi:10.1016/J.HEALUN.2012.03.015 9386683

[14] Duong SQ , Zhang Y , Hall M , Hollander SA , Thurm CW , Bernstein D , *et al*. Impact of institutional routine surveillance endomyocardial biopsy frequency in the first year on rejection and graft survival in pediatric heart transplantation. Pediatr Transplant 2021;25:e14035. doi:10.1111/petr.14035 34003559

[15] Keogh AM , Billingham ME , Schroeder JS . Rapid histological changes in endomyocardial biopsy specimens after myocarditis. Br Heart J 1990;64:406‐408. doi:10.1136/hrt.64.6.406 2271352 PMC1224822

[16] Dec GW Jr , Fallon JT , Southern JF , Palacios IF . Relation between histological findings on early repeat right ventricular biopsy and ventricular function in patients with myocarditis. Br Heart J 1988;60:332‐337. doi:10.1136/hrt.60.4.332 3190962 PMC1216581

[17] Dec GW , Fallon JT , Southern JF , Palacios I . “Borderline” myocarditis: an indication for repeat endomyocardial biopsy. J Am Coll Cardiol 1990;15:283‐289. doi:10.1016/s0735-1097(10)80050-6 2299069

[18] Aretz HT , Billingham ME , Edwards WD , Factor SM , Fallon JT , Fenoglio JJ Jr , *et al*. Myocarditis. A histopathologic definition and classification. Am J Cardiovasc Pathol 1987;1:3‐14. 3455232

[19] Richardson P , McKenna WJ , Bristow M , Maisch B , Mautner B , O'Connell J , *et al*. Report of the 1995 World Health Organization/International Society and Federation of Cardiology Task Force on the definition and classification of cardiomyopathies. Circulation 1996;93:841‐842. doi:10.1161/01.CIR.93.5.841 8598070

[20] The jamovi project . jamovi (version 2.5). 2024. https://www.jamovi.org. Accessed 1 May 2024

[21] Baritussio A , Schiavo A , Basso C , Giordani AS , Cheng CY , Pontara E , *et al*. Predictors of relapse, death or heart transplantation in myocarditis before the introduction of immunosuppression: negative prognostic impact of female gender, fulminant onset, lower ejection fraction and serum autoantibodies. Eur J Heart Fail 2022 Jun;24:1033‐1044. doi:10.1002/ejhf.2496 35377503

[22] Mason JW , O'Connell JB , Herskowitz A , Rose NR , McManus BM , Billingham ME , *et al*. A clinical trial of immunosuppressive therapy for myocarditis. The myocarditis treatment trial investigators. N Engl J Med 1995;333:269‐275. doi:10.1056/NEJM199508033330501 7596370

[23] Frustaci A , Chimenti C , Calabrese F , Pieroni M , Thiene G , Maseri A . Immunosuppressive therapy for active lymphocytic myocarditis: virological and immunologic profile of responders versus nonresponders. Circulation 2003;107:857‐863. doi:10.1161/01.cir.0000048147.15962.31 12591756

[24] Caforio ALP , Kaski JP , Gimeno JR , Elliott PM , Laroche C , Tavazzi L , *et al*. Endomyocardial biopsy: safety and prognostic utility in paediatric and adult myocarditis in the European Society of Cardiology EURObservational Research Programme Cardiomyopathy and Myocarditis Long‐Term Registry. Eur Heart J 2024;45:2548‐2569. doi:10.1093/eurheartj/ehae169 38594778 38594778

[25] Ferreira VM , Schulz‐Menger J , Holmvang G , Kramer CM , Carbone I , Sechtem U , *et al*. Cardiovascular magnetic resonance in nonischemic myocardial inflammation: expert recommendations. J Am Coll Cardiol 2018;72:3158‐3176. doi:10.1016/j.jacc.2018.09.072 30545455

[26] Zainal H , Rolf A , Zhou H , Vasquez M , Escher F , Keller T , *et al*. Comparison of diagnostic algorithms in clinically suspected viral myocarditis: agreement between cardiovascular magnetic resonance, endomyocardial biopsy, and troponin T. J Cardiovasc Magn Reson 2024;26:101087. doi:10.1016/j.jocmr.2024.101087 39191369 PMC11565394

[27] Francone M , Chimenti C , Galea N , Scopelliti F , Verardo R , Galea R , *et al*. CMR sensitivity varies with clinical presentation and extent of cell necrosis in biopsy‐proven acute myocarditis. JACC Cardiovasc Imaging 2014;7:254‐263. doi:10.1016/j.jcmg.2013.10.011 24560214

[28] Power A , Hernandex NB , Dipchand AI . Rejection surveillance in pediatric heart transplant recipients: critical reflection on the role of frequent and long‐term routine surveillance endomyocardial biopsies and comprehensive review of non‐invasive rejection screening tools. Pediatr Transplant 2022;26:e14214. doi:10.1111/petr.14214 35178843

[29] Patel JK , Kpbashigawa JA . Should we be doing routine biopsy after heart transplantation in a new era of anti‐rejection? Curr Opin Cardiol 2006;21:127‐131. doi:10.1097/01.hco.0000210309.71984.30 16470149

[30] Sinphurmsukskula S , Ariyachaipanicha A , Siwamogsathamb S , Thammanatsakula K , Puwanantb S , Benjacholamasc V , *et al*. Endomyocardial biopsy and prevalence of acute cellular rejection in heart transplantation. Transplant Proc 2021;53:318e323‐318e323. doi:10.1016/j.transproceed.2020.08.014 33041079

[31] Committee W , Drazner MH , Bozkurt B , Cooper LT , Aggarwal NR , Basso C , *et al*. 2024 ACC expert consensus decision pathway on strategies and criteria for the diagnosis and Management of Myocarditis: a report of the American College of Cardiology Solution Set Oversight Committee. J Am Coll Cardiol 2025;85:391‐431. doi:10.1016/j.jacc.2024.10.080 39665703

[32] Angelini A , Crosato M , Boffa GM , Calabrese F , Calzolari V , Chioin R , *et al*. Active versus borderline myocarditis: clinicopathological correlates and prognostic implications. Heart 2002;87:210‐215. doi:10.1136/heart.87.3.210 11847154 PMC1767046

[33] Basso C , Calabrese F , Angelini A , Carturan E , Thiene G . Classification and histological, immunohistochemical, and molecular diagnosis of inflammatory myocardial disease. Heart Fail Rev 2013;18:673‐681. doi:10.1007/s10741-012-9355-6 23096264

[34] De Gaspari M , Larsen BT , d'Amati G , Kreutz K , Basso C , Michaud K , *et al*. Diagnosing myocarditis in endomyocardial biopsies: survey of current practice. Cardiovasc Pathol 2023;64:107494. doi:10.1016/j.carpath.2022.107494 36415008

